# Editorial: Molecular and cellular logic of cerebral cortex development, evolution, and disease

**DOI:** 10.3389/fnana.2023.1242684

**Published:** 2023-07-07

**Authors:** Maria Teresa Dell'Anno, Luciano Conti, Marco Onorati

**Affiliations:** ^1^Fondazione Pisana per la Scienza ONLUS, San Giuliano Terme, Italy; ^2^Department of Cellular, Computational and Integrated Biology, University of Trento, Trento, Italy; ^3^Department of Biology, University of Pisa, Pisa, Italy

**Keywords:** cortical development, cortical evolution, astrogenesis, minicolumn, cortico-thalamic circuit

Among central nervous system structures, the cerebral cortex is widely recognized as the hub of higher cognitive functions that distinctly characterize humans. With its intricate network of connections, bewildering variety of cell types, and peculiar multilaminated structure, the cerebral cortex has undergone a dramatic evolution over time accompanied primarily by an increase in proportions in relation to body size. Indeed, the gyrification process is a result of the expansion of novel classes of progenitor cells, especially the outer radial glia, that produce and provide migration guidance for upper-layer cortical neurons, many of which are characteristic of the most evolved species and define the neocortex, underlining its recent phylogenetic origin.

This Research Topic aims to present a comprehensive overview of the latest advancements in cerebral cortex development. Special attention is given to the role of the molecular mechanisms that coordinate its assembly, the involvement of non-neuronal cells in its development and in the acquisition of cognitive abilities, and the evolutionary factors influencing its cytoarchitecture.

In humans, the neural tube closes ~30 days post-conception, and the neocortex forms at the rostral end of the neural tube, through the migration of neurons originating from proliferative regions near cerebral ventricles of the telencephalon (Sidman and Rakic, 1973; Marin and Rubenstein, 2003). Migration ensures that layers generate in an inside-out fashion. Therefore, layer 1 is the most external and the first to be generated, followed by deep infragranular layer 5 and 6 neurons, then granular layer 4 neurons, and, eventually, layer 2 and 3 neurons (Cadwell et al., 2019). The ultimate identity of a cortical neuron and its definitive allocation are attained through the coordinated activation of crucial transcription factors (Kast and Levitt, 2019). However, the transcriptional profile *per se* may not be sufficiently informative, as translation into proteins may be delayed, depending on mRNA stability, localization, and editing (Zahr et al., 2018; Park et al., 2022). Cremisi and Vignali focus on the post-transcriptional control attributed to RNA-binding proteins (RBPs) and microRNAs during corticogenesis. Both RBPs and microRNAs operate as translational repressors exerting control over various processes, including neural proliferation and differentiation (Franzoni et al., 2015), layering (Shu et al., 2017), and plasticity (Letellier et al., 2014). The authors summarize how microRNAs (i.e., mir-3607, mir- 122, and mir-137) may exert a heterochronic effect in cortical neuron maturation by refining the translated protein's temporal appearance. This suggests that microRNAs also have a role in the evolution of the mammalian brain, facilitating increases in neural progenitor cells or regulating differentiation and migration (Tomasello et al., 2022), two processes that eventually sustain the enlargement of cortical areas that anatomically differentiate gyrencephalic species.

The question of whether proliferative niches persist in the adult brain has been extensively studied, with the detection of mitotic events in the adult hippocampus of rodents (Alvarez-Buylla and Lim, 2004). This area originates from the medial pallium, which has been thoroughly investigated in mammals and in anamniotes due to its involvement in learning and navigation (Salas et al., 2006; Sotelo et al., 2016). Amphibians, the only extant anamniote tetrapods, express conserved transcription factors deemed necessary for mammalian hippocampal development (Moreno et al., 2004; Lust et al., 2022; Woych et al., 2022). However, little is known about the evolution of the medial pallium transcriptional profile in amniotes and anamniotes. In a comparative analysis of the expression pattern of conserved markers in the amphibian *Xenopus laevis* and in the amniote *Trachemys scripta*, Jiménez and Moreno reported that, despite cytoarchitectural differences in the layering of the medial pallium, expression of the gene *Prox1* and transcription factors Er81 and Lmo4 was shared with the mammalian dentate gyrus, thus providing evidence of a common genoarchitectonics supporting the functional involvement in memory tasks.

Another hallmark of superior brain function is reciprocal connections between the cerebral cortex and the thalamus. The cortico-thalamic and thalamo-cortical circuits elaborate essential tasks such as wakefulness, sensory processing, learning and memory, plasticity, and consciousness. Disorders that affect higher brain functions, including schizophrenia, bipolar disorder, and autism spectrum disorders, impact this system. Angulo Salavarria et al. summarize cortico-thalamic formation, starting with the prosomeric model of neurodevelopment (Rubenstein et al., 1994; Puelles et al., 2013). The cerebral cortex and the thalamus operate as a single unit, and the establishment of their reciprocal connections was observed in the human embryo at ~7.5/8 post-conceptional weeks. Various models have been used to study the molecular and cellular mechanisms of cortico-thalamic development. In parallel to animal models, which remain fundamental for unveiling neural network establishment, the authors critically discuss advanced *in vitro* platforms, e.g., brain organoids and assembloids derived from human pluripotent stem cells. These innovative tools have substantial potential for basic research in brain development and dysfunctions. Furthermore, *in silico* techniques are used to mimic composite brain circuitry, simulating realistic inputs/outputs and clarifying neuron interaction in complex networks.

The arrangement of minicolumns is another crucial aspect of cortical structure that has undergone evolutionary changes across different species (Buxhoeveden and Casanova, 2002). Morphologically defined as strings of interconnected neurons extending radially across layers 2–6 (Rakic, 1988), minicolumns are the elemental processing unit of the neocortex and have been detected in diverse cortical areas. The iterative repetition of these structures is thought to be fundamental to the neocortical expansion that has characterized brain size augmentation with evolution and the increase in computational power (Rakic, 1995, 2008). Here, Wallace et al. explore features of minicolumns present in the primary visual cortex (V-1) in five mammalian orders: human and non-human primates (*Homo sapiens, Pan troglodytes*, and *Gorilla gorilla*), rodents (*Cavia porcellus, Mus musculus*, and *Rattus rattus*), Eulipotyphla (*Erinaceous europeus*), Artiodactyla (*Sus scrofa*), and Carnivora (*Mustela putorius*). The authors describe a spatial arrangement of minicolumnar bundles of the primary visual cortex (V-1) in linear or branched strings with variable intra-layer length, density, and intracolumnar distance, depending on the species (Wallace et al.). In general, V-1 minicolumns spanned from the base of layer 3 to the white matter in all great apes including humans, and carnivores, whereas other mammalian orders had a diverse structure made of repeating modules or microcolumns with a shorter layer extension and more irregularity in the spatial patterning. There was a strong association between the abundance of minicolumns and visual acuity, thus indicating the existence of a relationship between a numerical parameter in cortical cytoarchitecture and an indicative function of the computational power.

Several observations have supported the notion that non-neuronal cells, specifically astroglia, may affect cortex development and evolution. Findings included unique characteristics of primate astroglia, implying a potential contribution to cortical processes (Oberheim et al., 2009; Zhang et al., 2016; Vasile et al., 2017; Falcone and Martinez-Cerdeno, 2023). The study by Degl'Innocenti and Dell'Anno summarized prevalent astrocyte differences between mice and humans in an overview that starts with cortical astrogliogenesis and includes morphological and functional differences. Compared with rodents, human astrocytes have a higher degree of complexity in size, morphology, and extension of intercellular interactions, all aspects that reflect their increased capacity in fostering synaptic transmission and increasing mouse cognitive capacities upon engraftment (Han et al., 2013). Human astrocytes are generated from the ventricular and subventricular zones, similarly to the mouse. However, human-specific glial precursors have been identified in the basal or outer radial glia cells, the prominent gliogenic capacity of which is responsible for the thickening of the cortex and the development of convolutions (Rash et al., 2019).

In summary, the findings in this Research Topic offer a comprehensive overview of factors participating in cortex development and key divergences that have led to the acquisition of distinctive species-specific features. These elements collectively contribute to our understanding of the intricate processes shaping the cortex and its evolutionary trajectory. The data in these five articles, in conjunction with studies outside this Research Topic, should provide clues for uncovering the logic behind the vast heterogeneity of the human cerebral cortex, not only to reveal underlying mechanisms in neurological or psychiatric disorders but also to disclose the neurobiological elements conferring the uniqueness, multifaceted talents, and capacities of the human brain.

## Author contributions

All authors listed have made a substantial, direct, and intellectual contribution to the work and approved it for publication.
